# Hospital Is Not the Home: Lessons From Implementing Remote Technology to Support Acute Inpatient and Transitional Care in the Home in the United States and United Kingdom

**DOI:** 10.2196/58888

**Published:** 2024-10-11

**Authors:** Matt Wilkes, Annabel Kramer, Juliana Pugmire, Christopher Pilkington, Benjamin Zaniello, Nicole Zahradka

**Affiliations:** 1 Best Buy Health Boston, MA United States

**Keywords:** telemedicine, implementation science, hospital-to-home transition, remote patient monitoring, digital health, transition of care, accuracy, acceptability

## Abstract

The COVID-19 pandemic, patient preference, and economic opportunity are shifting acute care from the hospital to the home, supported by the transformation in remote monitoring technology. Monitoring patients with digital medical devices gives unprecedented insight into their physiology. However, this technology does not exist in a vacuum. Distinguishing pathology from physiological variability, user error, or device limitations is challenging. In a hospital, patients are monitored in a contrived environment. Monitoring at home instead captures activities of daily living alongside patients’ trajectory of disease and recovery. Both settings make for “noisy” data. However, we are familiar with hospital noise, accounting for it in our practice and perceptions of normal. Home monitoring as a diagnostic intervention introduces a new set of downstream consequences, dependent on device, cadence of collection, adherence, duration, alarm thresholds, and escalation criteria. We must accept greater ambiguity and contextualize vital signs. All devices balance accuracy with acceptability, so compromises are inevitable and perfect data should not be expected. Alarms must be specific as well as sensitive, balancing clinical risk with capacity for response. By setting expectations around data from the home, we can smooth the adoption of remote monitoring and accelerate the transition of acute care.

## Introduction

Remote patient monitoring (RPM) has been evolving for over 60 years. In 1961, NASA developed the technology to monitor the Project Mercury astronauts’ electrocardiography, temperature, and respiration rate in flight [1]. Since then, RPM has developed in parallel with health care technology, but the SARS-CoV-2 pandemic was an inflection point in demand. Significant volumes of acute and transitional care moved from in person to virtual, and from the hospital to the home [2]. Continued government support via telehealth reimbursement and the Centers for Medicare and Medicaid Services Acute Hospital Care at Home Waiver has been a critical enabler, but so has provider and patient preferences: remote care can ease access, and care at home for many conditions is seen as preferable to a clinic or hospital visit [3,4]. Economically, it is an enticing proposition with the potential to reduce cost of care, free up inpatient capacity, and reduce readmissions [5]. Remotely monitoring patients with digital medical devices can give unprecedented insight into their physiology, and RPM is now a core part of programs as health care delivery transitions to the home [6].

These devices measure familiar vital signs such as heart rate, respiratory rate, oxygen saturation, and temperature. However, the advantages of acute care at home, particularly patients’ ability to perform their activities of daily living, mean that data from the home are not the same as those from the hospital. Indeed, in this viewpoint, we argue that their superficial similarity mandates a conscious adjustment in our expectations and practice of monitoring. Clinicians, researchers, and innovators building on data gathered from home monitoring must understand the substrate of their work. A planned implementation, including appropriate education of all involved, is key to the adoption of new technologies in health care [7]. Without educating clinicians about the differences between hospital and home monitoring, and giving them strategies to practice effectively, they can quickly lose confidence in the technology. We have implemented intermittent and continuous home monitoring in acute care settings (predominantly hospital at home and oncology) in 21 US health systems (22,264 patients) between July 2019 and 2024, and 22 UK National Health Service trusts (11,862 patients) between December 2017 and July 2024. The median (IQR) length of stay between patients first transmitting vital signs to discharge was 3.9 (1.9-12.9) days in the United States and 7.3 (4.6-12.2) days in the United Kingdom. Our implementations have typically included the provision and configuration of proprietary and third-party remote monitoring devices, electronic medical record integration, care pathway design, staff training, logistics, and program troubleshooting and evaluation. In addition, our US nursing triage service has provided direct monitoring for 5515 patients in hospital-at-home and acute oncology settings since December 2021. In this viewpoint, we highlight some underappreciated differences between home and hospital monitoring and share our best practices, ranging from the philosophical to the tactical, to smooth implementation and make effective use of this promising technology.

Hospitals are highly contrived environments. Trained practitioners collect vital signs with patients being awake, deliberately positioned, and motionless [8]. The architecture of the hospital floor, the social roles of clinician and patient, and the ingrained rituals of care dictate the interaction. In contrast, monitoring in the home is more naturalistic. Patients live and act according to their condition, social role, resources, and environment. This lack of restriction is, in many ways, the “point” of care in the home, key to its therapeutic intent and the richness of its monitoring data [9,10]. Both the hospital and the home make for “noisy” data. However, we are more familiar with hospital noise, so we subconsciously account for it in our practice and our perceptions of “normal” vital signs [11,12]. When there is ambiguity, we can more easily physically examine the patient. The noise of the home is novel, and the patient is out of our immediate reach so we cannot visually contextualize it. We must actively familiarize ourselves with this new approach to monitoring if we are to use it to its full potential.

## Accuracy Versus Acceptability

**Figure 1:**
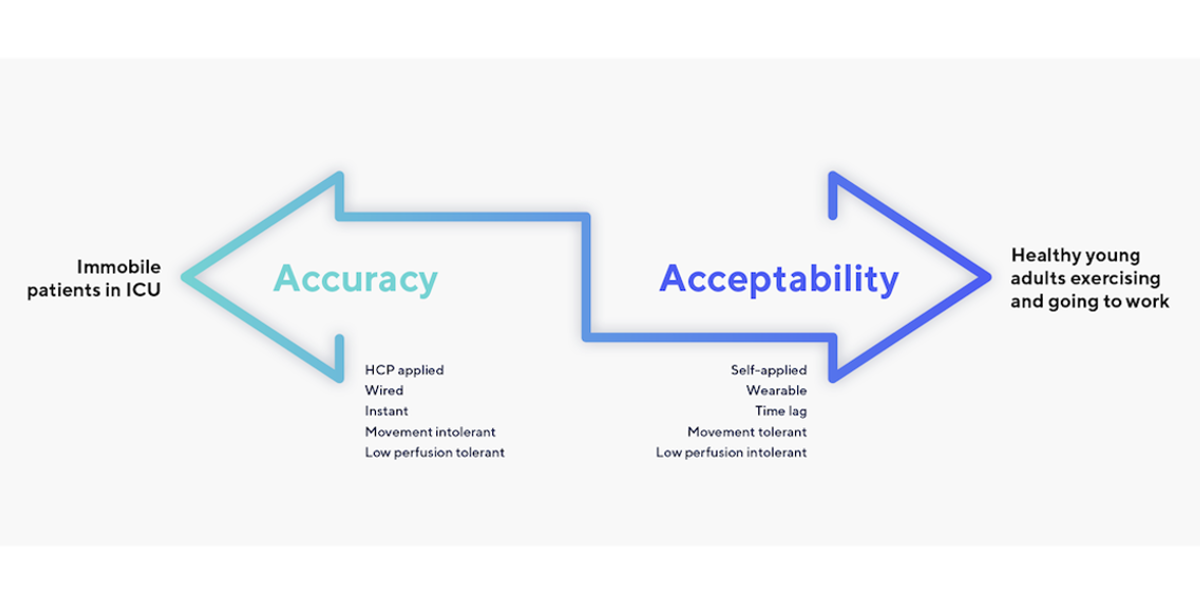
The accuracy-acceptability spectrum. HCP: health care provider; ICU: intensive care unit.

Care in the home takes measurement out of clinicians’ hands. The patient collects their own vital signs or passively wears the equipment unsupervised. Both carry the possibility of user error. We must also accept compromises in the technology itself. Many hospital monitoring devices are heavy, expensive, complex, wired, and unintuitive. They are not suitable for the home. Home monitoring equipment must be untethered, less obtrusive, and more intuitive for patient adherence [13]. Trading accuracy for adherence is the price of home monitoring, and a balance must be struck between accuracy and acceptability depending on the population and goals of the monitoring (Figure 1). For example, a finger pulse oximeter is not practical to wear constantly. So, we gather oxygen saturation and heart rate from watches, armbands, or patches instead. These devices reflect light from the skin instead of transmitting it through the digital arteries of the finger like a hospital probe. The available signal is reduced, rendering them more vulnerable to motion artifact, high ambient light, or changes in skin perfusion [14]. When validated in laboratory conditions, both finger probes and wearables can perform equally well, but the laboratory is not the home either.

## Continuous Versus Intermittent

Vital sign monitoring in the home can be continuous (via stick-on patches or armbands), intermittent (eg, finger pulse oximeters or blood pressure cuffs), or both. Continuous monitors collect data passively (the patient just wears them), while intermittent monitors require a patient to initiate a reading. Continuous monitoring has historically been the province of critical care and the operating room. When untethered continuous monitoring became possible, the ease of passive data collection and the superficial reassurance of “ICU-style monitoring” made it an appealing solution for acute care in the home. However, its utility is an evolving question: it remains unclear whether (1) the advantages of passive data collection and reduced interobserver variability outweigh the challenges of managing high data volumes and (2) whether the opportunity to detect deterioration more rapidly exceeds the consequences of incidental findings that subsequently prove innocuous [12,15,16].

Continuous monitoring requires effective handling of large volumes of data [17]. Data are typically aggregated into windows of time (eg, “median SpO_2_ over the last hour”) to make them digestible and facilitate transmission to the electronic health record (EHR). Longer windows of aggregation mean fewer data points to triage with greater suppression of outliers and variability (more specificity and less sensitivity), which helps mitigate noise from activities of daily living but can mask brief but clinically significant periods of deterioration. Aggregation also introduces time lags compared to spot monitoring [18]. These time lags mean that discrepancies between continuous and intermittent data streams frequently arise, even if both are functioning correctly, with the potential for loss of confidence in the technology if not well understood by users.

**Figure 2:**
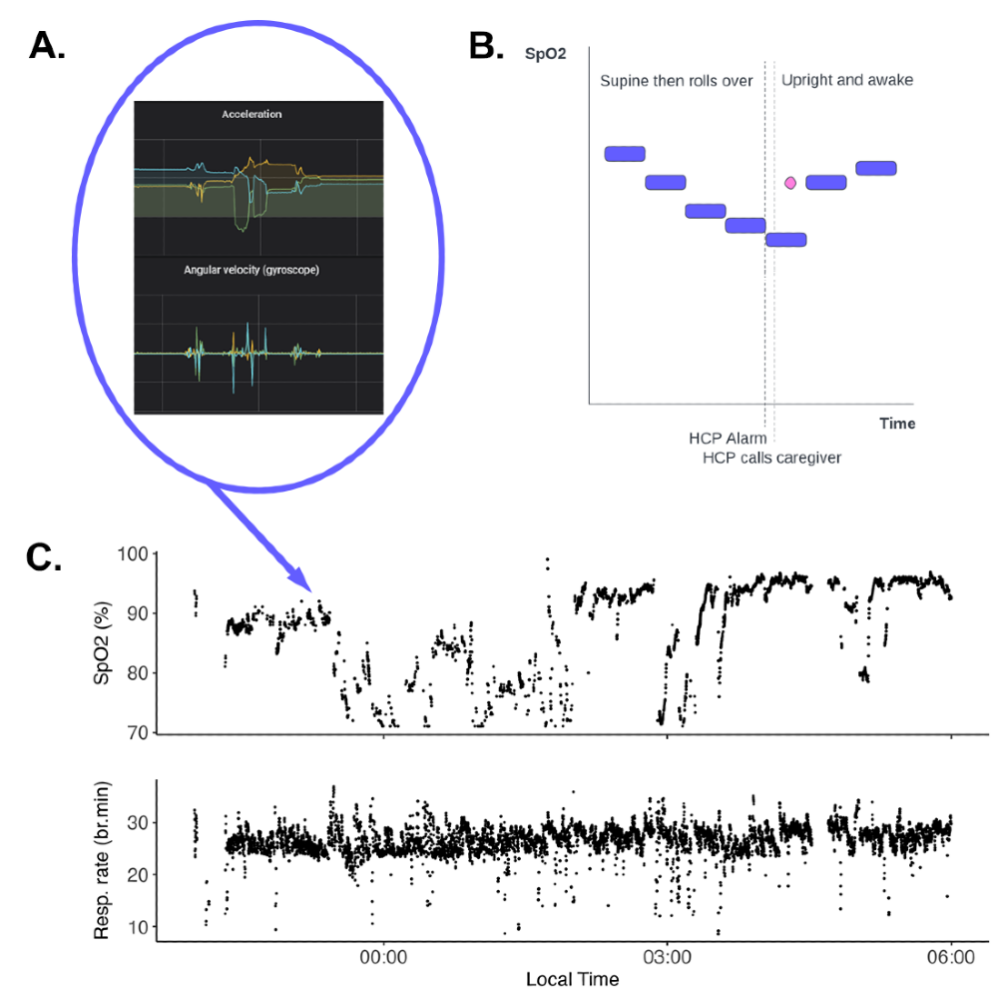
(A) Data from motion-sensing accelerometers and gyroscopes; (B) the basis of the discrepancy between continuous and spot data: the blue bars represent aggregated continuous data from the wearable, lagging behind the spot monitoring by the finger probe (pink dot); (C) raw monitoring data illustrating 3 episodes of hypoventilation and hypoxia, after a postural change recorded by the motion-sensing accelerometers and gyroscopes. HCP: health care provider; Resp.: respiratory; SpO2: oxygen saturation.

Figure 2 illustrates this scenario. The patient was an older adult man in a US hospital-at-home program, with cellulitis of the lower limb. He had been sent home with a continuous monitor (Current Health Gen 2; Best Buy Health), and his main caregiver had independently purchased an over-the-counter finger pulse oximeter. The raw monitoring data (Figure 2C) indicated 3 episodes of hypoventilation and hypoxia, after a postural change that was visible on the motion sensors (Figure 2A). Figure 2B illustrates the misunderstanding that followed. The continuous data were aggregated into medians over time for ease of recording in the EHR (Figure 2B; the blue bars represent this aggregation). During sleep, the patient rolled onto one side and their oxygen saturation levels decreased. The nursing triage service called his caregiver, who woke the patient, sat him up, and encouraged him to take deep breaths. The patient’s saturation levels rapidly rose again. The patient’s caregiver also asked the patient to put on their finger probe and the patient’s oxygen saturation levels were normal (Figure 2B; pink dot). The continuous monitor, on a longer time aggregation, lagged and still showed hypoxia before normalizing. The caregiver queried this apparent discrepancy, which resulted in an investigation. On close of examination of the continuous data, it appeared the patient had sleep apnea. This was confirmed by a subsequent sleep study. The continuous monitor was dismissed as inaccurate, with the more familiar finger probe treated as the “reference.” Yet, it was the continuous monitor, with its capacity for detailed review of raw signal data, that identified the pathology. This example also illustrated the potential for incidental findings with continuous monitoring, leading to downstream investigations that, in the case of this nonagenarian patient, were unlikely to affect his overall prognosis.

## Interpreting Variability

**Figure 3:**
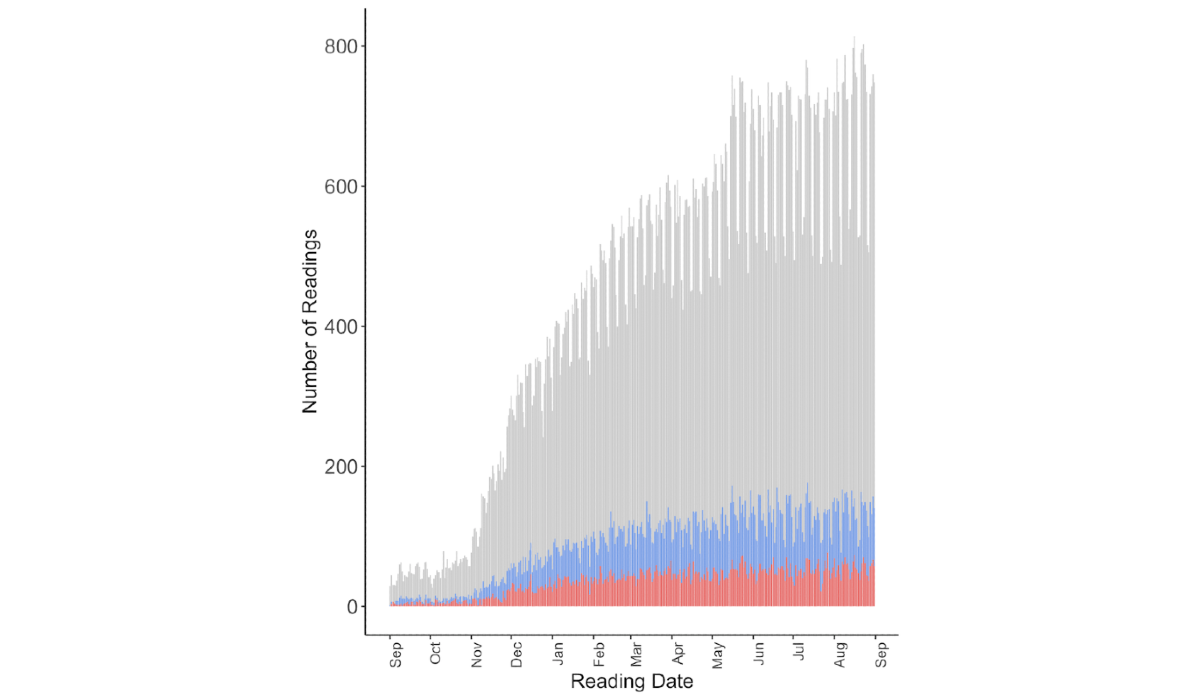
Variations in systolic and diastolic blood pressure readings during the rollout of an Food and Drug Administration 510(k)–cleared cuff in the home (5418 patients). The gray bars are the total number of daily readings (n=159,645), the blue bars are readings where the systolic variance from the previous day is >20 mm Hg (median 20%, IQR 19%-22% of readings), and the red bars are readings where the diastolic variance is >20 mm Hg (median 8%, IQR 7%-10% of readings). For reference, a systematic review found clinical blood pressure measurement to vary from true resting blood pressure, from –23.6 to +33 mm Hg for systolic blood pressure and from –14 to +23 mm Hg for diastolic blood pressure, due to patient, device, procedural, and observer factors, indicating that this problem is not unique to the home.

When a monitoring device records an unexpected value, either in the hospital or in the home, we must parse whether it represents genuine clinical change, natural variation, user behavior, user error, device error, or a combination of the above (Figure 3) [19]. It begs the question of what is a “normal” or expected reading. Humans exhibit inter- and intraindividual variability, even when healthy and at rest. We have predictable patterns of variation in vital signs, for example, circadian rhythms and menstrual cycles, as well as unpredictable patterns based on activity and internal and external stimuli [20,21]. These are further complicated by our trajectories of disease or recovery [22]. In the hospital, variation is suppressed by intermittent spot monitoring under contrived circumstances, or its cause is obvious: for example, a tachycardia might be due to a patient being suddenly awoken, just having taken medication, standing up, or receiving bad news. A data stream from the home lacks this context, so our challenge becomes distinguishing between variability and pathology. In intermittent monitoring, there may be too few data points to do this easily, and in continuous monitoring, there may be too many [18]. Nonetheless, trends in variability are becoming increasingly important indicators of health and disease [22,23]. These are readily suppressed in the hospital but better preserved in the home.

## Monitoring Successfully in the Home: Lessons Learned

### Overview

We believe that monitoring a patient in their own environment during acute and transitional care can be less intrusive and more informative than in the hospital. However, we need to implement home monitoring collaboratively and iteratively, being mindful of the technology and the needs of the service, while managing the expectations of all involved [24]. The following recommendations are based on our experience.

### Choose and Test Technology to Achieve a Specific Goal

It is important to select technology based on the program’s goals, rather than simply adopting what is available, previously purchased, or the norm on the hospital floor. Then, the technology should be actively studied on how it performs remotely. Regulatory clearance (eg, Conformité Européenne [CE] marking or Food and Drug Administration 510(k) clearance) is the minimum bar for any clinical device, but the laboratory validation studies required for clearance give little indication of how well it will perform in the home. Pragmatic testing in the context in which the technology will be used is essential to ensure it will be fit for purpose. This includes ensuring equitable provision for patients with low levels of digital literacy or access [25]. Challenges such as the provision of infrastructure or subsidization of access are more easily addressed on a governmental level. However, technology providers can work to design accessible standards, develop specific training programs, incorporate translation services into platform communications, and ensure that cultural sensitivities are respected [26]. Building in free-roaming cellular connectivity to devices, rather than relying solely on patients’ own internet connections, is an effective way to allay concerns about incurred costs. In all cases, user experience testing is key to validating assumptions about the target population. For example, one might have assumed that older patients would struggle with remote monitoring technology. In a comparison between 316 patients aged over 75 years and 541 younger patients using remote monitoring equipment, the older group did indeed rate themselves as more likely to avoid technology or delay adoption (82% vs 56%) and scored the technology as marginally less easy to use (ease-of-use score 5.5 vs 6.2) [27]. However, the older patients exhibited higher adherence to wearable use (95.3% vs 93.3%; *P*<.001) and equivalent adherence to remote monitoring tasks such as blood pressure measurement. The obvious implication was that patients should not be excluded because of age, but the more nuanced finding was that patient education should be focused on building confidence in the technology as much as skill acquisition.

Once a candidate technology is chosen, how it will work in the broader ecosystem should be considered, including interoperability, cybersecurity, and the flow of aggregated data into the EHR. Specifically, this may include procedures to annotate or amend data flowing from the home into the EHR if the data are missing or erroneous. This is not always straightforward. As Figure 1 illustrated, there is a compromise between accuracy and acceptability: no wearable device will ever be completely accurate all the time and may occasionally transmit incorrect or artifactual data to the medical record. This is also true of manual measures in the hospital, which have considerable interobserver variability, but these are more of a “cultural norm,” and in the home, there are usually no additional measures to corroborate the data from the wearable [19]. One approach is to annotate remotely collected vital signs with a measure of confidence that the vital signs accurately reflect the patient’s physiology, based on the strength and regularity of the waveform data, the degree of interference (eg, from movement), and the percentage of data available in each window of time.

### Set Expectations Early and Educate Throughout the Program

Before implementing remote monitoring, it is important to educate hospital clinicians to expect different and more variable data. Gaps in continuous data from the home are normal. Perfect accuracy or coverage is not expected (or necessary) to make good decisions, even in acute care. Our nursing team has been able to safely monitor 5515 patients at home with a median (IQR) adherence to continuous monitoring devices of 79% (77%-81%; defined as the percentage time data transmitted over length of stay)—in other words, with approximately 20% of data “missing.” This remains the case even for the most vulnerable populations: in a cohort of 40 patients with hematological malignancies receiving chimeric antigen receptor T-cell (CAR-T) therapy managed acutely at home (median length of stay 14.7, IQR 10.4-27.6 days), overall adherence to continuous monitoring devices was 79% (IQR 68%-88%) [28]. Median (IQR) survey completion was 50% (7.7%-80%) and blood pressure adherence was 95% (69.8%-100%). A total of 25 patients developed cytokine release syndrome while at home but were safely managed, despite incomplete data. We advise repeated training sessions as clinicians gain familiarity with this new stream of data. Patients and caregivers should be trained to use the equipment properly, accounting for their knowledge, motivation, skills, systems, and behaviors [13]. If the collected vital signs are visible to patients and carers, then a sufficient degree of education and annotation should be insured so they can react constructively to their data without undue concern [25].

### Prescribe a Dose of Monitoring

It is recommended to move away from the hospital practice of applying “standard monitoring.” Instead, clinicians should be encouraged to consider home monitoring as a diagnostic intervention with downstream clinical, operational, and financial consequences. An appropriate “dose” of monitoring should be prescribed to capture meaningful change. A monitoring prescription might be used for intermittent or continuous data capture, specifying a bespoke cadence of data collection, adherence, duration, alarm thresholds, escalation, and discharge criteria. The prescription may need to be adjusted over time to reflect the patient’s clinical trajectory. As home monitoring matures, we can evolve dose-response curves to titrate monitoring across different use cases. This dovetails with the previous point regarding education and setting expectations. In the outpatient CAR-T program described above, 30 patients triggered a total of 670 clinical alarms (0.5 per patient-day) over 4 months of monitoring [28]. These alarms prompted 1102 calls by monitoring nurses to patients, 63% of which were during nonclinic hours. The alarms were set to trigger when the median of a particular vital sign, aggregated over 30 or 60 minutes, breached a set threshold. When a 15-minute aggregation window was tried in an analogous program, 6 patients triggered a total of 436 alarms in 1 month without a notable difference in outcomes to the first program.

### Individualize Alarms

**Figure 4:**
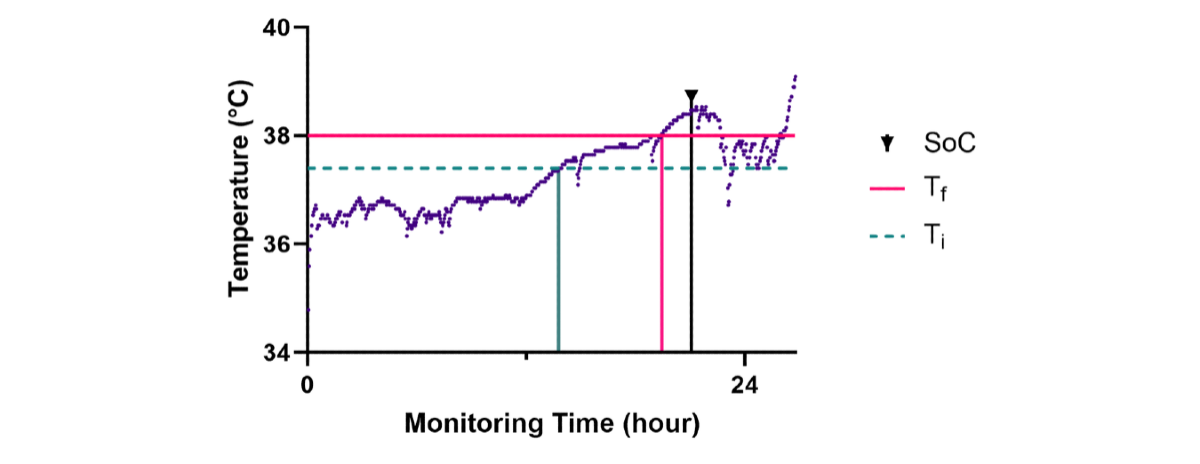
Axillary temperature plot of a patient receiving immunotherapy in the 24 hours before a diagnosis of CRS. The green dashed line represents an alarm of individual baseline temperature plus 2 SDs (Ti), and the pink line represents a fixed alarm at 38 °C (Tf). The arrow mark is the time the nurse recorded CRS onset (SoC). The use of a continuous monitoring system led to earlier detection than standard nursing care, as did an individualized threshold compared to a fixed threshold of 38 °C. CRS: cytokine release syndrome; SoC: standard of care.

It is beneficial to individualize alarms in the home. In the hospital, a patient’s baseline vital signs, especially their variability, will be suppressed as they rest in bed. In the home, there will be more variation. We advise using alarm thresholds and trends tailored to individual patients and based on their clinical condition, risk, and social circumstances, rather than generic hospital “normal” values [29]. Their baseline values should be established early in their home stay, and then the focus should be on deviations from that baseline, rather than absolute values [30]. This recommendation is perhaps best illustrated with an inpatient example, where a “gold standard” comparator (standard nursing care) can be used. In a study of 28 patients receiving CAR-T therapy in the hospital with a remote monitoring device worn as a “black box,” a simple alarm set at baseline temperature plus 2 SDs allowed the detection of temperature disturbances indicative of cytokine release syndrome a median of 184 (IQR 53-312) minutes earlier than standard care [31]. By comparison, the use of a fixed temperature threshold only improved detection by a median of 22 (IQR –43 to 113.5) minutes without a significant increase in specificity (Figure 4 [32]). Using trend-based measures is also an approach to deal with the variability in the peripheral devices described in Figure 3. While Figure 3 concerned blood pressure, in a systematic review and meta-analysis of 75 studies, Niven et al [33] demonstrated strikingly broad limits of agreement between peripheral and central measures of temperature, especially among adults with fever (–1.44 to 1.46 °C). In other words, peripheral measures of temperature in 2 patients with the same core temperature could vary by ~3 °C. This supports moving to a trend-based diagnosis from the fixed hospital threshold of 38 °C in febrile illness.

### Balance Sensitivity, Specificity, Risk, and Capacity to Respond

**Figure 5:**
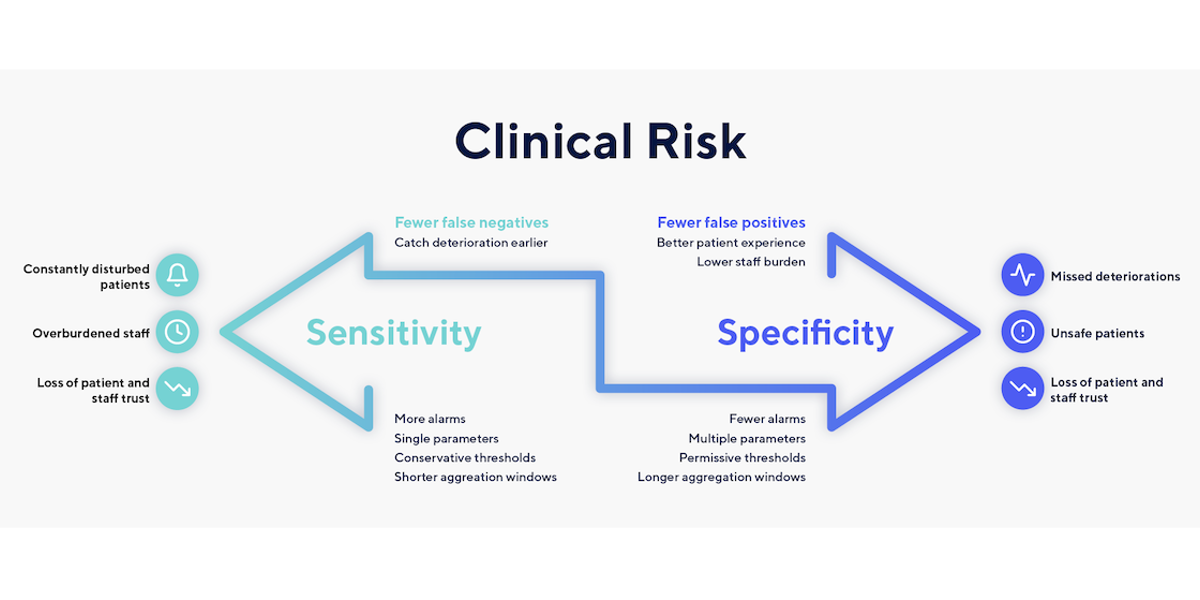
Balancing sensitivity and specificity with clinical risk.

In the hospital, we can use alarms that are purely sensitive (high risk of false positives and low risk of false negatives). Alarm fatigue is an issue, but it is straightforward to examine the patient following an alarm [34]. In contrast, a false alarm in the home may entail waking the patient or caregiver or dispatching a paramedic unnecessarily. We must therefore introduce more specificity. However, if we push either sensitivity or specificity too far, there will be a loss of trust from the patients and clinicians, financial penalties, and degradation of clinical care, as in the oncology example above (Figure 5).

We must find a balance of sensitivity and specificity, while being mindful of our clinical risk tolerances and our capacity to respond to alarms. All decisions should be based on new and variable data. Accepting this balance allows us to give patients space to recover in their own home and grants us a rich stream of diagnostic information. It is the crux of the mindset shift to home monitoring. It also requires establishing a feedback loop to differentiate useful and nonuseful alarms, adjusting the balance of sensitivity and specificity accordingly. This titration can be challenging, as there may be a gap between an alarm sounding and its utility being understood. Furthermore, those monitoring the data, assessing the patient, and modifying alarms might all be different individuals in different locations. In all cases, escalation criteria should be agreed in advance, being mindful that clinicians receiving the referral may not be intimately familiar with the nuances we have described above. We suggest erring toward sensitive settings at the start and increasing specificity as confidence grows.

### Conduct “Rounds” Regularly on Patients

It is important to not rely solely on alarms. Instead, teams should conduct “rounds” on a virtual care patient’s data as often as their condition warrants to become familiar with the patterns in their vital signs. Our monitoring team conducts a “round” on patients every 4-6 hours, as they might on the hospital floor. A “round” involves reviewing the patient’s vital signs and any asynchronous communications. This helps nurses establish patterns that differentiate the routine from the pathological, as well as picking up issues like incorrect device wear. Nurses also use surrogates for examining a patient. For example, compensatory changes in other vital signs, measures of motion, postural change, speed of vital sign recovery following activities of daily living, and adherence to monitoring all paint a picture of a patient in the home. These observations can then be fed back to patients’ care teams during multidisciplinary “huddles” [35]. Our nurses have anecdotally reported that hypoxia and rising temperatures are most frequently spotted early by rounding, particularly when the patient’s health care providers have chosen alarms that maximize specificity. As one nurse described:

During rounding on one hospital at home patient, I noted her SpO2 had been gradually decreasing - but no other vital sign was doing the same [and she had not yet triggered an alarm]. I called her and she was short of breath…she no idea what to do with herself. I called the responsible clinician and was told to place her supplementary oxygen on immediately. I did this and stayed on with her until we had a sustained acceptable spot check (and she wasn’t huffing and puffing). She was educated by site the next day on supplementary oxygen use.

### Acknowledge the Stressors and Set Appropriate Standards of Care

Above all, we must recognize the learning curve and acknowledge the initial uncertainty and stress for care teams with this new stream of data. Clinicians should be monitored as they develop meaningful relationships with their RPM patients [36]. With time, they will develop confidence to not disturb patients, such as the patient referred to in Figure 2, with every fluctuation in vital signs—even in the acute setting. While desirable, this is also medicolegally vulnerable position, as the clinicians would opt not to respond to values traditionally considered “abnormal” in hospital, to give patients the space to recover. Malpractice is judged as deviation from an accepted standard of care. While there are now national figures for escalation and mortality rates (6.2% and 0.5%, respectively, in the United States), there are no standards of care for responding to data from the home [2,37]. If we default to hospital standards of care (rapid response to every change in vital signs and alerting senior staff), we either undermine care at home by overcontacting patients or risk a monitoring care team being in legal jeopardy for appearing to be unduly lax. So, we must set new standards for care at home, supporting clinicians in their decisions, building facilities for them to easily document their judgment calls, and constructing achievable escalation pathways around them. Standards and governance need to develop alongside a program as it evolves, with experienced clinicians mentoring those joining the program. Given all the limitations and subtleties we have described, we advise caution in entrusting too much triage, too early, to machine learning alone.

### Consider Data Ownership, Evaluation, and Labeling Before Deployment

There is often insufficient clarity around data ownership in an RPM program. There is a need to establish early on what data belong to the patient, the technology provider, and the health system [25]. This should be considered for both identifiable and deidentified data. This is particularly pertinent in the context of program evaluation and health care research. The most useful data are typically those labeled with symptoms or behavior by the patient, with quality and completeness by the technology provider, and with clinical context by the health system. If all 3 cannot be physically and legally united, then evaluators and researchers would not hold all pieces of the puzzle.

## Conclusions

We all recognize that hospitals are often not ideal healing environments due to their architecture and processes (bright lights, other patients, vital checks, and overhead codes) and that shifting acute care to the home may positively impact patient satisfaction, clinical outcomes, and health care economics. However, the home can be as noisy as the hospital, only for different reasons. Patients’ vital signs are products of these environments, and as clinicians and researchers, we must interpret them in their proper context. Monitoring patients in the home where they fall ill, recover, and thrive is a powerful tool to understand health and disease, but differences to hospital monitoring must be understood, expected, acknowledged, and—we believe—celebrated in the next 25 years of RPM’s evolution.

## Abbreviations

CAR-T: chimeric antigen receptor T-cell
CE: Conformité Européenne
EHR: electronic health record
RPM: remote patient monitoring
